# Ischemic preconditioning enhances energy supply during frequency speed kick test in Taekwondo athletes: A randomized crossover study

**DOI:** 10.1371/journal.pone.0341780

**Published:** 2026-02-03

**Authors:** Ziyue Ou, Liang Yang, Dongwei Xie, Huaiyuan Zhu, Xiquan Weng, Guoqin Xu

**Affiliations:** 1 College of Martial Arts, Guangzhou Sport University, Guangzhou, China; 2 Guangdong Engineering Polytechnic, Guangzhou, China; 3 School of Sports Training, Guangzhou Sport University, Guangzhou, China; 4 College of Exercise and Health, Guangzhou Sport University, Guangzhou, China; 5 Guangdong Provincial Key Laboratory of Physical Activity and Health Promotion, Guangzhou Sport University, Guangzhou, China; Sheffield Hallam University, UNITED KINGDOM OF GREAT BRITAIN AND NORTHERN IRELAND

## Abstract

**Purpose:**

This study aimed to examine the effect of ischemic preconditioning (IPC) on energy supply during sport-specific tests in male taekwondo athletes. To our knowledge, this is the first investigation of IPC’s efficacy in enhancing energy supply in a taekwondo context, underscoring its potential to optimize performance in combat sports.

**Methods:**

Sixteen male taekwondo athletes completed a randomized crossover trial comparing an IPC condition (220 mmHg) with a sham condition (20 mmHg). Performance was evaluated via the frequency speed kick test (FSKT), and energy system contributions were quantified via the PCr–La–O₂ method. Two-way repeated-measures ANOVA and a generalized linear mixed model were used to analyze the data.

**Results:**

IPC significantly increased the total number of kicks in each FSKT group (98.4 ± 6.6 vs. 94.0 ± 5.5, p = 0.049, d = 0.72; 94.4 ± 7.7 vs. 89.3 ± 5.5, p = 0.036, d = 0.77; 93.1 ± 7.5 vs. 86.8 ± 6.3, p = 0.015, d = 0.90) and the aerobic energy supply (294.09 ± 50.35 vs. 270.44 ± 49.30, P = 0.031, d = 0.47). The postexercise blood lactate clearance rate was greater in the IPC trial (14.07 ± 4.94 vs. 9.14 ± 6.84, p = 0.02, d = 0.81), despite no differences in glycolytic or phosphagen contributions (49.32 ± 13.11 vs. 43.00 ± 12.25, p = 0.055).

**Conclusion:**

IPC appears to enhance taekwondo performance by improving aerobic metabolism and accelerating lactate clearance, thereby promoting synergistic interactions between anaerobic and aerobic energy systems and improving energy supply. Notably, the performance benefits of IPC became more pronounced as exercise duration increased, suggesting a cumulative ergogenic effect during prolonged high-intensity activity.

## Introduction

Ischemic preconditioning (IPC) is an intervention involving brief cycles of ischemia and reperfusion that activate the body’s intrinsic protective mechanisms [1]. Since Murry’s first description of IPC in 1989, it has proven effective at reducing long-term ischemic injury (e.g., myocardial ischemia) in clinical settings [2]. In recent years, IPC has garnered increasing attention within the field of sports science and has been integrated into sports training practices [3–6]. This involves applying sufficiently high pressure (a common pressure for the lower limbs is 220 mmHg) to the limb prior to exercise training to achieve local limb blood flow occlusion [7], which lasts for 5 minutes, followed by a 5-minute rest, with multiple repetitions to stimulate the protective mechanisms of IPC [1,8]. Accumulating evidence indicates that IPC can enhance athletic performance in various contexts. For example, applying IPC improved cyclists’ incremental exercise performance, yielding a 3% increase in maximal oxygen uptake (VO₂max) [9], a key indicator of aerobic capacity [10,11]. IPC has also been reported to improve anaerobic performance: in Wingate anaerobic tests, it increases the average power output [3,12], and similar benefits have been observed in sports such as basketball [7], swimming [8], and judo [13]. Mechanistically, IPC triggers the release of vasoactive and metabolic factors such as nitric oxide and adenosine, which can accelerate the clearance of lactate and H^+^ and thereby enhance high-intensity endurance. IPC-induced ischemia also generates mild oxidative stress that activates adaptive pathways (e.g., NF-κB) and upregulates the expression of antioxidant enzymes (e.g., superoxide dismutase), increasing cellular tolerance to subsequent oxidative bursts [14]. In an animal experiment involving male Wistar rats, IPC was shown to maintain ATP synthesis capacity and improve mitochondrial respiratory function [15]. It has been hypothesized that IPC promotes mitochondrial uptake of acetyl-CoA [8], facilitating the Cori cycle to maintain lactate levels within a controllable range and sustain ATP resynthesis, which may explain improvements in maximal exercise capacity [16]. IPC may additionally act by opening mitochondrial ATP-sensitive K^+^ channels (mK_ATP_), thereby optimizing energy metabolism and reducing energy expenditure during exercise [17]. Collectively, these findings suggest that IPC can enhance athletic performance through improved metabolic efficiency. However, research on IPC’s ergogenic effects, especially in sport-specific scenarios, remains limited, indicating a need for further investigation.

Taekwondo, an Olympic combat sport, features a best-of-three match structure with 2-minute rounds and 1-minute recovery intervals. This format necessitates a unique interplay between anaerobic and aerobic energy systems to support rapid, high-intensity kicking sequences and facilitate efficient recovery [18,19]. Despite IPC’s performance benefits in sports such as cycling, swimming, and judo via metabolic modulation [8,9,13], its efficacy in taekwondo has not been thoroughly studied. Previous studies on IPC in the context of taekwondo have focused primarily on its impact on performance [20,21], without investigating the specific characteristics of energy metabolism changes associated with the unique demands of taekwondo competition schedules. Notably, no studies have detailed how IPC influences the unique energy metabolism profile of the intermittent, high-intensity competition format of taekwondo. This gap is important given taekwondo’s distinct physiological demands: athletes must deliver technical, explosive kicks over multiple rounds with limited recovery. To address this shortfall, the present study is the first to evaluate IPC in a taekwondo-specific context by using the frequency speed kick test (FSKT) and quantifying energy system contributions via the PCr–La–O₂ method. This approach allows us to investigate not only whether IPC can improve taekwondo performance but also how it alters the underlying aerobic and anaerobic energy supply characteristics. By doing so, we aim to provide novel insights that could inform training and performance optimization in this sport.

The FSKT is a widely used assessment of taekwondo-specific performance capacity [22]. This method offers a convenient, rapid, and relatively accurate reflection of the specific athletic abilities of taekwondo athletes [23]. When combined with the rapid part method of oxygen debt (PCr-La-O_2_) [24], it allows for a comprehensive analysis of energy metabolism during specialized tests [25], thereby elucidating the unique physical fitness characteristics of taekwondo athletes. Building on the FSKT methodology and its associated energy metabolism analysis, this study posits that IPC may enhance the athletic performance of taekwondo athletes by modulating energy metabolism. The objectives of this study were twofold: (1) to determine whether IPC can improve taekwondo athletes’ performance in a sport-specific test and (2) to assess whether IPC can increase energy availability during taekwondo-specific exercise, characterizing any shifts in energy system contributions. Through this research, we aim to provide scientific guidance for taekwondo practitioners on the application of IPC in both daily training and precompetition contexts, with the goal of enhancing their competitive results and overall athletic performance.

## Materials and methods

### Subjects

To determine the sample size, an a priori power analysis was conducted via G*Power 3.1, which was based on expected effect sizes from prior studies on IPC in combat sports and intermittent exercise [13,26]. Assuming a medium-to-large effect size (f = 0.40) for the primary outcome (total number of kicks in the FSKT), a power of 0.80, and an alpha of 0.05 for a two-way repeated-measures ANOVA with two trial conditions and three measurement points, a minimum sample size of 14 participants was needed. To account for potential dropouts, 16 male taekwondo athletes were recruited. A post hoc power analysis confirmed a power of 0.97 (effect size f = 0.45, partial η² = 0.169) for the FSKT total kick outcome, indicating sufficient statistical power. The inclusion criteria for the experimental subjects were as follows: (1) adult male athletes aged 18 years or older; (2) taekwondo athletes classified as national second-class or above (having placed in the top three provincial professional taekwondo championships or the top eight national professional taekwondo championships); (3) consistent and unified training over the past three months; (4) no prior ischemic preconditioning; and (5) competitive taekwondo athletes. The exclusion criteria were as follows: (1) supplementation with creatine; (2) acute or chronic illnesses, including anxiety, depression, cardiovascular diseases, and metabolic disorders; (3) engaging in vigorous physical activity within 48 hours prior to the experiment; or (4) consuming alcohol or caffeine within 24 hours before the experiment.

The recruitment period for subjects was from March 1 to March 31, 2024. The subjects were required to visit twice, with a minimum interval of one week between visits, and each visit involved one subject receiving the intervention. The participants provided written informed consent to participate in this study. The formal trial intervention period was scheduled from April 15 to June 1, 2024. Before each experiment, the researchers will inquire about the participants’ physical condition again via phone or in person, and inform them of the precautions to be taken before the experiment, ensuring that the participants are in a healthy state prior to the experiment. The primary reasons for not conducting clinical registration prior to participant recruitment are as follows: 1) IPC is a safe and noninvasive intervention that has been widely applied in exercise training practices; 2) this study emphasizes the effects of IPC as a preexercise intervention on athletic performance rather than its application in the clinical treatment of diseases. The experiment has completed post hoc clinical registration (ClinicalTrials.gov ID: NCT07170774, 11/09/2025). The authors confirm that all ongoing and related trials for intervention are registered.

The research protocol received approval from the Ethics Review Committee of Guangzhou sport university (ID number: 2023LCL-81) and adhered to the guidelines established in the Declaration of Helsinki.

### Experimental protocol of the study

The experiment employs a single-blind randomized crossover controlled design. The participants were required to complete two experimental sessions, with a minimum interval of one week between each session. In the two sessions, the thigh pressure is applied at values of 220 mmHg and 20 mmHg. The order of these sessions is randomized. The 220 mmHg pressure is designated the IPC, whereas the 20 mmHg pressure is designated the sham trial (SHAM). Nevertheless, it would be a limitation if the subjects can determine the type of intervention by perceiving different pressures. Independent statistician A, who was not involved in the recruitment of subjects, generated the allocation sequence via R software (version 4.3.2). A block randomization design was employed (with a block length of 4) to ensure a balanced distribution of the two intervention sequences (A-B or B-A) throughout the recruitment period. A random seed number (20240904) was set to ensure reproducibility. We strictly implemented allocation concealment via the sequentially numbered, opaque, sealed envelope (SNOSE) method. The envelopes were prepared by research assistant B and were kept by another research assistant C. For each eligible subject recruited, the researcher contacted research assistant C to open the next sequential envelope and learn the assigned sequence. Owing to the necessity of researcher involvement in pressure adjustment, a double-blind design was not feasible. However, participants were blinded to the trial type (IPC vs. SHAM) to minimize expectancy effects. Moreover, the experimental subjects were informed that the purpose of this study was to compare the effects of different pressures on athletic performance to reduce placebo effects.

Each experiment consists of three parts:

Part 1: Preparation Before the Experiment and Pressurization Intervention.

Upon arrival at the test site, the experimental subjects rested quietly for 10 minutes. Blood pressure is measured, body composition is assessed, and a heart rate belt is worn to monitor heart rate throughout the process. Fingertip peripheral blood was collected to measure blood lactate levels. The subjects also wore a portable cardiopulmonary exercise testing device (used to collect data on gas metabolism during the test) and IPC equipment. After the participants sat quietly for 5 minutes to stabilize their breathing, a 40-minute pressurization intervention (20 mmHg or 220 mmHg) was conducted. Following this intervention, subjects continue to lie flat and rest for an additional 5 minutes, after which fingertip peripheral blood lactate is measured at the end of 3 minutes.

Part 2: Warm-Up Activities Before the Test.

After resting, the subjects engage in a 10-minute warm-up activity, which includes 5 minutes of jogging (with the heart rate controlled at approximately 120 beats per minute) followed by three sets of 20-second medium-intensity kicks (with a 1-minute interval between sets and a heart rate controlled at 140--160 beats per minute) and flexible stretching. The pattern of kicks is designed to resemble the FSKT method to familiarize experimental subjects with the exercise testing process. After the warm-up, the subjects rested for 5 minutes before fingertip blood samples were collected prior to the specific test. The kicking action of FSKT is the most commonly used technical maneuver among professional taekwondo athletes during competitions, and there is no learning effect associated with it.

Part 3: Taekwondo Specific Test.

**Fig 1:**
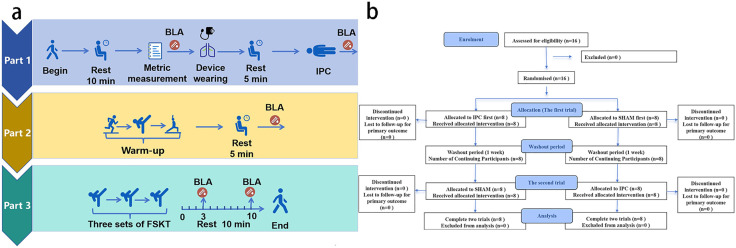
Experimental flow diagram and CONSORT 2025 flow diagram. a: Experimental flow diagram, BLA: blood lactate, IPC: ischemic preconditioning. b: CONSORT 2025 flow diagram.

The experimental subjects simulate a taekwondo competition system by conducting three sets of FSKT tests, with a 1-minute rest between sets. After completing the specific test, breathing gas is continuously collected from the subjects until the 6-minute mark, and blood lactate levels are measured at the end of the 3rd minute [27,28] and the end of the 10th minute post-exercise. The experimental process is illustrated in Fig 1a. The CONSORT 2025 flow diagram is shown in Fig 1b.

### Basic indicator measurement methods

Heart rate was monitored via a Polar H10 device (Finland). Blood pressure measurements were taken in both the sitting and standing positions via an OMRON model HEM-1020 monitor. It is essential that the blood pressure value on the experimental day remains below 140 mmHg (to prevent accidents caused by hypertension in experimental subjects). Body composition analysis was conducted via the InBody 370 Body Composition Analyzer (Korea). Blood samples were collected via finger prick tests, and blood lactate concentrations were analyzed via a Biosen C-line analyzer (Biosen C-line, EKF Diagnostics). The clearance rate of blood lactate following the exercise test was calculated via the following formula [29]:


$$y=\frac{{{L\text{ac}}^{-}}_{\text{1}}-{{L\text{ac}}^{-}}_{\text{2}}}{{{L\text{ac}}^{-}}_{\text{1}}}\times \text{1}\text{0}\text{0}\%$$

*Lac*^*-*^_1_ (mmol/L) refers to the concentration of blood lactate measured previously, whereas *Lac*^*-*^_2_ (mmol/L) denotes the concentration of subsequent blood lactate measurement [29].

### IPC

Under normal oxygen conditions, the experimental subjects were placed in a supine position to conduct the IPC trial. Blood pressure cuffs (Theratools, Lower Extremity Kit, Cuff width: 10 cm) were applied to the left and right thighs of the subjects, which were positioned close to the inguinal region. The cuff on the right leg was inflated to 220 mmHg, whereas the cuff on the left leg remained at 0 mmHg for 5 minutes. The pressure in the right leg cuff was subsequently reduced to 0 mmHg, and the left leg cuff was subsequently inflated to 220 mmHg for another 5 minutes, thereby completing one cycle. This procedure was repeated for a total of 4 cycles, amounting to 40 minutes [4,7,30]. In the SHAM trial, the pressure was altered from 220 mmHg to 20 mmHg. To confirm effective ischemia during the IPC protocol, a multidimensional assessment was employed. Operators, certified as Chinese medical practitioners, use a stethoscope to confirm the absence of anterior tibial artery pulse sounds during cuff inflation to 220 mmHg [31–34]. Additionally, palpation verified the absence of pulsation at the posterior tibial artery (medial malleolus). Visual inspection was used to assess skin pallor in the toes and plantar region within 1 minute of pressure application, with pallor indicating successful occlusion. Postrelease, the return of normal skin color (within 10–15 s) and a subjective sensation of warmth confirmed reperfusion. Subjective reports of numbness or tingling in the feet were also recorded. Effective ischemia was confirmed when at least three of these criteria (absent pulse, pallor, and subjective sensations) were met, ensuring that the intervention achieved the intended occlusion-reperfusion effect.

### Taekwondo-specific sports performance test

The frequency speed kick test (FSKT) evaluates taekwondo-specific performance by measuring the ability to deliver rapid, high-intensity kicks [22,23,35–37]. The version of FSKT used in this study is the intermittent version (5 x 10s/10s intervals, FSKT_mult_) [22]. The participants performed three sets of FSKT, with a 1-minute rest between sets, simulating the structure of a formal taekwondo competition. Each set consisted of five 10-second rounds of maximal-effort roundhouse kicks to professional electronic protective equipment designed for Taekwondo competitions (WT professional version, China), alternating between legs, with 10-second rest intervals between rounds (total set duration: 90 seconds). The electronic scoring system automatically recorded valid kicks on the basis of an impact force exceeding a preset threshold (minimum of 50 Newtons), ensuring accurate and objective measurement of the number of kicks per round and total kicks per set.

### Energy metabolism analysis method

Energy metabolism during FSKT was quantified via the PCr-La-O₂ method, which estimates contributions from phosphagen, glycolytic, and aerobic systems on the basis of oxygen uptake and lactate accumulation [24,25]. Respiratory gas collection was performed via a portable cardiopulmonary exercise testing device (JAEGER Oxycon Mobile, Germany). The device was calibrated daily according to the manufacturer’s protocol, involving a 30-minute warm-up, volume calibration with a 3-L syringe, and gas calibration using a reference gas mixture (15% O₂, 5% CO₂) (Calibrate the tests between each subject). Gas collection began 5 minutes before the IPC or SHAM intervention to establish baseline oxygen uptake, continued throughout the warm-up and FSKT, and extended for 6 minutes post-exercise to capture excess postexercise oxygen consumption (EPOC). Blood lactate samples were collected immediately before the FSKT and at 3 and 10 minutes post-exercise.

The process for metabolic gas collection and testing was as follows:

Upon arrival, after completing the basic indicator tests, blood lactate samples are collected. The subjects then don the portable cardiopulmonary exercise testing device, initiate the equipment, and gather gas metabolism data. After a 5-minute period of quiet sitting to allow breathing to stabilize, they undergo a 40-minute intervention (either IPC or SHAM). Following the intervention, they rest for 5 minutes before engaging in a 10-minute warm-up activity, which includes low-intensity exercise, dynamic stretching, and three sets of 20-second moderate-intensity kicking exercises, with the heart rate maintained between 140 and 160 beats per minute. After an additional 5-minute rest, a blood lactate sample is collected immediately before the commencement of the FSKT test. The subjects then completed the FSKT test as needed, with continuous respiratory gas collection throughout the process. Gas collection continues for 6 minutes following the test, and blood lactate sampling occurs at the 3rd minute after the completion of the three sets of FSKT tests. Finally, the energy supply is calculated on the basis of the gathered gas metabolism data and blood lactate data.

The calculation formula is as follows:


$$\text{phosphagen}\;\text{energy}\;\text{supply}\;\text{part}={\text{VO}}_{\text{2PCR}}\;(\text{ml})\times \text{Ee}(\text{J}/\text{ml})$$

$$\text{Energy}\;\text{supply}\;\text{for}\;\text{the}\;\text{glycolysis}\;\text{group}={{\text{Lac}}^{-}}_{\text{accum}}\;(\text{mM})\times \alpha \left({\text{O}}_{\text{2}}-\text{LA}\right)\;(\text{ml}/\text{kg}/\text{mmol}/\text{L})\times \text{weight}\;(\text{kg})\times \text{Ee}(\text{J}/\text{ml})$$

$$\text{Aerobic}\;\text{metabolism}\;\text{energy}\;\text{supply}\;\text{part}=\;{\text{VO}}_{\text{2accum}}(\text{ml})\times \text{Ee}(\text{J}/\text{ml})$$

VO_2PCR_ refers to the rapid component of VO_2_ following exercise, with an energy equivalent (Ee) of 21.131 J/ml [25]. Blood lactate accumulation (Lac^-^_accum_) during specific tests is calculated as the peak blood lactate level after exercise minus the blood lactate level immediately prior to the specific test. For this study, the blood lactate level measured at the end of the third minute post-exercise was considered the peak lactate value [27,28]. The oxygen‒lactate conversion coefficient (α(O_2_-LA)) was set at 3.0 ml/kg/mmol/L [38]. In addition, the accumulated oxygen uptake (VO_2accum_) during exercise is defined as the actual oxygen uptake minus the resting oxygen uptake. The resting oxygen uptake (VO_2_) is standardized at a fixed value of 4.5 ml/kg/min [25].

After the three sets of FSKT tests, VO_2_ can be categorized into three components: a fast component, a slow component, and a steady component (denoted as VO_2(t)_). The fast component primarily occurs within the first three minutes following the specific test. Consequently, the postexercise VO_2_ can be divided into the VO_2(t)_ of the initial three minutes and the VO_2(t)_ of the subsequent three minutes. The exported gas metabolism data were further analyzed via Origin 2021 software. The VO_2_ data from the last three minutes are selected for trend line fitting, and the resulting trend line is extrapolated back to the first three minutes to determine the slow component at each time point within this interval. Finally, the fast component is calculated by subtracting the extrapolated slow component from the actual VO_2_ recorded during the first three minutes. The definite integral of the oxygen uptake rate curves for each component was computed via Origin 2021 to quantify the oxygen uptake accumulated for each respective component.

### Data analysis

The cumulative total oxygen consumption at each stage of the experiment was calculated via Origin 2021 software. Statistical analyses were conducted via SPSS 25.0 and R 4.4.3, with the Shapiro‒Wilk test used to assess the distribution of variables such as heart rate, blood lactate, blood oxygen saturation, lactate clearance, exercise performance, oxygen consumption, and energy metabolism. For the intertrial comparisons of the blood lactate clearance rate, peak heart rate, and energy metabolism variables that conformed to a normal distribution, paired sample t tests were employed. For the total scores of each group in the FSKT tests, as well as the average heart rate and oxygen consumption variables across stages, which either conformed to or approximated a normal distribution and required repeated testing, two-way repeated-measures ANOVA was conducted. Mauchly’s test of sphericity was performed, and for those not meeting the sphericity assumption, corrections were applied via the Greenhouse–Geisser method. The effect size (partial η²) was calculated, with values interpreted as trivial when 0 ≤ partial η² ≤ 0.01, small when 0.01 < partial η² ≤ 0.06, medium when 0.06 < partial η² ≤ 0.14, and large when partial η² > 0.14. All two-way repeated measures ANOVAs were corrected for multiple comparisons via the Bonferroni correction, and the effect sizes for post hoc simple comparisons were defined via Cohen’s standards [39]: Cohen’s d value was calculated, with d = 0.2 indicating a small effect, d = 0.5 indicating a medium effect, and d = 0.8 indicating a large effect. For the intertrial comparison of the first-round kick test variables that do not conform to a normal distribution and require repeated measurements, a generalized linear mixed model (GLMM) with the gamma distribution family and log link function was employed. The fixed effects included the interaction term of trial type (Trial) and time point (Timepoint), whereas the random effects consisted of a random intercept for subject number (NO). The main effects and interactions were assessed via the Type III Wald chi-square test, and post hoc comparisons were conducted via unadjusted pairwise comparisons (emmeans). A conservative correction was subsequently performed via the Bonferroni correction. For the intertrial comparison of blood lactate levels at various stages, the same GLMM approach with the gamma distribution family and log link function was utilized. Model fitting was performed via the bobyqa optimization algorithm (with the maximum number of iterations set at 200000), and post hoc tests involved comparisons of trial conditions within time points (Tukey correction). The criterion for statistical significance was a p value of less than 0.05.

We conducted a post hoc power analysis via G*Power 3.1.9.2 to determine the sample size. In this study, the results of the intertrial effect test for the total number of kicks in each FSKT test showed a partial η² value of 0.169, leading to a calculated effect size f of 0.45. We subsequently revalidated the sample size by selecting the repeated-measures ANOVA (within factor) in the F test, setting α at 0.05 and the power at 0.95, with the trials divided into 2 and the number of measurements set at 3. Ultimately, the calculated power is 0.97, significantly exceeding the basic requirement of 0.8 [39], indicating that this study has a sufficient sample size. All the data are expressed as the means ± standard deviations ($\overset{―}{\text{X}}\;\pm \;\text{SD}$).

## Results

### Subjects

Sixteen male taekwondo athletes participated in this experiment. The basic information about the experimental subjects is presented in Table 1.

**Table 1:** Basic information of the study subjects (N = 16).

	Age (Y)	Height (cm)	Weight (kg)	Years of Training (Y)	BMI (kg/m^2^)	Body fat percentage (%)
$\overset{―}{X}\pm SD$	19.25 ± 1.29	180.43 ± 6.56	70.81 ± 7.20	6.63 ± 1.41	21.93 ± 2.21	12.13 ± 3.84

### Basic indicators and exercise intensity

**Fig 2:**
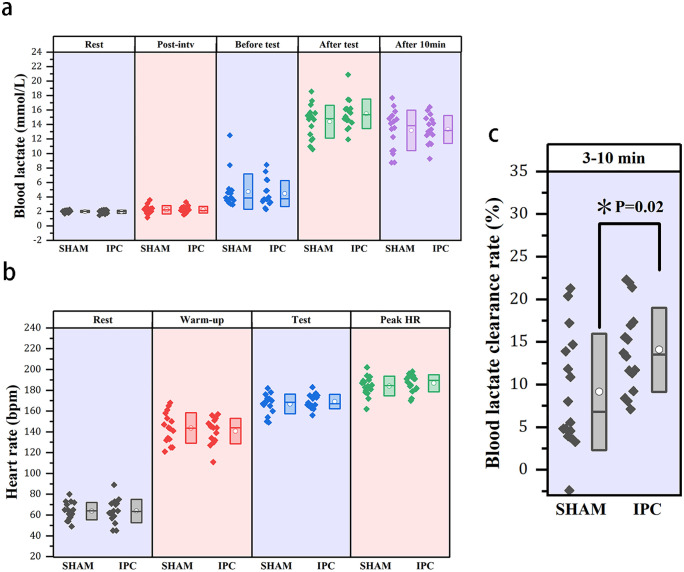
The box plot scatter diagram illustrates the basic indicators and sports intensity. a: Blood lactate levels during various states: at rest, postintervention, prior to the specific test, three minutes after the specific test, and ten minutes after the specific test. The label ‘Post-intv’ refers to blood lactate levels measured after the experimental intervention. The box in the figure represents the mean ± standard deviation, where the white dots within the box represent the average value, and the horizontal line denotes the median. Each scattered point corresponds to an individual sample value. b: The figure illustrates the average heart rates recorded during three distinct conditions: the quiet state, warm-up kicks, and specific tests. Peak HR: Peak heart rate during testing. c: The graph illustrates the blood lactate clearance rate from 3--10 minutes post-test. * indicates that the data conform to a normal distribution. A paired sample t test was employed, revealing a significant difference between the trials, with a p value of less than 0.05.

The results of the generalized linear mixed model indicated that the main effect between trials of blood lactate was not significant (χ²(1)=0.06, p = 0.806). Blood lactate concentrations at various stages were recorded as follows: at rest (1.99 ± 0.15 mmol/L vs. 1.95 ± 0.22 mmol/L, p = 0.806, d = 0.26, small effect); postintervention (2.22 ± 0.58 mmol/L vs. 2.23 ± 0.46 mmol/L, p = 0.931, d = 0.03, small effect); preexercise test (4.74 ± 2.44 mmol/L vs. 4.47 ± 1.80 mmol/L, p = 0.536, d = 0.12, small effect); 3 minutes post-exercise (14.39 ± 2.28 mmol/L vs. 15.49 ± 2.05 mmol/L, p = 0.318, d = 0.50, medium effect); and 10 minutes post-exercise (13.18 ± 2.80 mmol/L vs. 13.32 ± 1.94 mmol/L, p = 0.884, d = 0.06, small effect). Although no significant differences were observed between the trials (Fig 2a), the comparison of blood lactate tests at 3 minutes post-exercise revealed a medium effect, with the blood lactate concentration in the IPC trial showing an increasing trend. The results of the repeated-measures ANOVA for the average heart rate across stages indicated no significant main effect between trials (F = 0.04, p = 0.952, partial η² = 0). Simple comparisons between trials revealed no significant differences in average heart rate during the resting state (63.88 ± 11.28 bpm vs. 63.75 ± 8.36 bpm, p = 0.972, d = 0.01, small effect), warm-up phase (140.69 ± 12.27 bpm vs. 143.75 ± 14.73 bpm, p = 0.528, d = 0.22, small effect), or testing period (169.19 ± 6.86 bpm vs. 166.81 ± 9.47 bpm, p = 0.423, d = 0.28, small effect). Additionally, there was no significant difference in peak heart rate between trials (184.06 ± 9.42 bpm vs. 186.75 ± 8.26 bpm, p = 0.226, d = 0.30, small effect, paired samples t test), as illustrated in Fig 2b. These findings suggest that the intensities of the two trials are essentially consistent. Notably, the average peak heart rate during the test period was 187 beats per minute for the IPC trial and 184 beats per minute for the SHAM trial, both of which exceeded the high-intensity exercise threshold defined by the American College of Sports Medicine’s Guidelines for Exercise Testing and Prescription [40]. Furthermore, the lactate clearance rates from 3 to 10 minutes post-exercise demonstrated a significant difference in blood lactate clearance between the IPC and SHAM trials (14.07 ± 4.94% vs. 9.14 ± 6.84%, p = 0.02, d = 0.81, large effect). The IPC trial significantly increased the lactate clearance rate following the exercise test (Fig 2c).

### Results of accumulated oxygen uptake at each stage of the FSKT test

**Fig 3:**
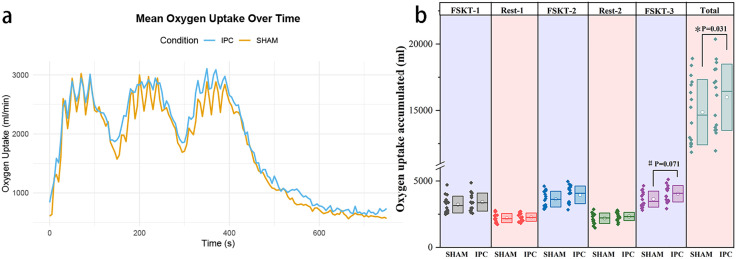
Changes and comparison chart of oxygen uptake. a: The x-axis represents time, and the y-axis represents oxygen uptake, including changes in oxygen uptake during the FSKT test and the 6-minute post-test. b: The boxplot scatter diagram illustrates the oxygen uptake accumulated at each stage of the special tests. *p < 0.05 indicates a significant difference when comparing IPC with SHAM; #p < 0.1 suggests a marginally significant difference between IPC and SHAM. The box in the diagram represents the mean ± standard deviation, the dots within the box denote the mean, and the horizontal line indicates the median. Each scatter point corresponds to the value of an individual sample.

The results indicated a significant main effect of time on cumulative oxygen uptake across repeated tests (F = 73.376, p = 0.000, partial η² = 0.71, large effect). Additionally, there was a marginally significant interaction effect between time and test, exhibiting a medium effect after the Greenhouse–Geisser correction was applied (F = 2.659, p = 0.095, partial η² = 0.081, medium effect). No significant main effect was found between trials, although a medium effect was noted (F = 1.98, p = 0.170, partial η² = 0.063, medium effect). The post hoc simple comparisons revealed no significant difference in cumulative oxygen uptake during the FSKT test for the first group (3220.01 ± 635.45 ml vs. 3407.72 ± 680.20 ml, p = 0.426, d = 0.28, small effect) or in the second group (3951.44 ± 664.05 ml vs. 3626.91 ± 595.01 ml, p = 0.156, d = 0.51, medium effect), despite a medium effect being observed. The IPC test results exhibited an increasing trend. The third set of FSKT tests demonstrated a marginally significant difference in cumulative oxygen uptake (4035.48 ± 622.43 ml vs. 3630.94 ± 600.18 ml, p = 0.071, d = 0.66, medium effect), along with a medium effect size and a marginally significant increase in cumulative oxygen uptake during the third IPC trial. There was no significant difference in cumulative oxygen uptake between the two intermittent periods (p > 0.05). Importantly, the total cumulative oxygen uptake throughout the entire exercise test was significantly greater in the IPC trial than in the SHAM trial (15988.57 ± 2495.49 ml vs. 14869.45 ± 2454.53 ml, p = 0.031, d = 0.45, medium effect). This finding suggests that as the duration of the exercise test increases, the cumulative effect of IPC on enhancing oxygen uptake becomes more pronounced. The average oxygen uptake over time for the SHAM and IPC test groups is illustrated in Fig 3a, while the cumulative oxygen uptake data for each stage are presented in Fig 3b.

### Results of the taekwondo-specific sports performance test

**Fig 4:**
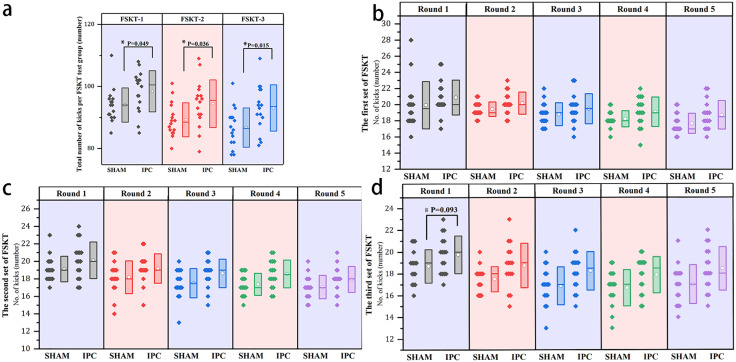
Special sports test performance boxplot scatter diagram. In the figure, Panel a presents the total number of kicks across each group of FSKTs, and a two-factor repeated measures analysis of variance was conducted to compare the effects of the two intervention methods. The P value represents the results of the simple effects between the two intervention methods. Panels b, c, and d present the results of each round of kicking for the first, second, and third groups of FSKT, respectively. *p < 0.05 indicates a significant difference when comparing IPC with SHAM; #p < 0.1 suggests a marginally significant difference between IPC and SHAM. The box in the figure represents the mean ± standard deviation, while the dots within the box signify the mean, and the horizontal line indicates the median. Each scattered point corresponds to the value of an individual sample.

The results of the FSKT tests on the experimental subjects indicate that the total number of kicks was relatively different (Fig 4a). The results of the two-factor repeated-measures ANOVA revealed a significant difference in the within-subject effects test (F = 35.525, P < 0.001, partial η² = 0.542, large effect), indicating that the total number of kicks in both the IPC and SHAM trials decreased over time. The interaction effect test between time and intervention method revealed no significant difference (F = 0.756, P = 0.443, partial η² = 0.025, small effect). The between-trial effects demonstrated a significant difference (F = 6.092, P = 0.02, partial η² = 0.169, large effect), with the total number of kicks in the IPC trial being significantly greater than that in the SHAM trial. The simple effects comparison between subjects revealed that in the first set of FSKT tests, the total frequency of the IPC trial was significantly greater than that of the SHAM trial (98.4 ± 6.6 vs. 94.0 ± 5.5, p = 0.049, d = 0.72, moderate to large effect). In the second set of FSKT tests, the IPC trial still showed a significant advantage (94.4 ± 7.7 vs. 89.3 ± 5.5, p = 0.036, d = 0.77, moderate to large effect). In the third set of FSKT tests, the IPC trial maintained a significant advantage in terms of the total number of kicks (93.1 ± 7.5 vs. 86.8 ± 6.3, p = 0.015, d = 0.90, large effect). The repeated measures analysis (based on GLMM) of the first-round scores for each set of FSKTs revealed that the main effect of trial type was marginally significant (χ²(1)=3.82, p = 0.0507), and the main effect of time point was also marginally significant (χ²(2)=5.00, p = 0.0823). However, the interaction effect between trial type and time point was not significant (χ²(2)=0.03, p = 0.9837). Specific pairwise comparisons at different time points indicated no significant differences in the first-round scores of the first set of FSKTs between trials (19.94 ± 2.93 vs. 20.88 ± 2.16, p-raw = 0.0507, p-adj = 0.101, d = 0.37, small effect). Similarly, the first-round scores of the second set of FSKTs were not significantly different between trials (19.13 ± 1.45 vs. 20.13 ± 2.09, p-raw = 0.0547, p-adj = 0.101, d = 0.54, medium effect). The first-round scores of the third set of FSKT showed a marginally significant difference between trials (18.69 ± 1.54 vs. 19.75 ± 1.73, p-raw = 0.0309, p-adj = 0.093, d = 0.65, medium effect), with a marginally significant improvement in the scores of the IPC trial. These findings suggest that the cumulative effect of IPC on enhancing athletic performance increases with prolonged exercise duration. Although we did not compare the differences between trials in other rounds, Fig 4b, 4c, and 4d show that the rate of decline in the mean performance per round during the IPC trials has slowed.

### The results of energy metabolism

The results of energy metabolism (Table 2) indicate that all variables conform to a normal distribution on the basis of the paired sample t test. Compared with the SHAM trial, the IPC trial significantly increased the aerobic energy supply (294.09 ± 50.35 kJ vs. 270.44 ± 49.30 kJ, p = 0.031). There was a marginally significant difference in the energy supply from the glycolytic system between the trials (49.32 ± 13.11 kJ vs. 43.00 ± 12.25 kJ, p = 0.055), with a marginal increase in the glycolytic energy supply in the IPC trial. Furthermore, there was no significant difference in the energy supply from phosphocreatine between the trials (42.79 ± 13.04 kJ vs. 42.99 ± 14.26 kJ, p = 0.968).

**Table 2:** Statistics of the energy metabolism of each energy supply system.

	SHAM	IPC	P	d
	$\overset{―}{X}\pm SD$	$\overset{―}{X}\pm SD$		
Phosphate energy supply (kJ)	42.99 ± 14.26	42.79 ± 13.04	0.968	0.02
Glycolysis energy supply (kJ)	43.00 ± 12.25	49.32 ± 13.11	0.055^#^	0.20
Aerobic energy supply (kJ)	270.44 ± 49.30	294.09 ± 50.35	0.031*	0.47

*p < 0.05 indicates a significant difference when comparing IPC with SHAM; ^#^p < 0.1 suggests a marginally significant difference between IPC and SHAM.

## Discussion

This study provides novel evidence that IPC enhances taekwondo-specific performance by improving aerobic metabolism and lactate clearance, thereby addressing a critical gap in the application of IPC to combat sports characterized by intermittent, high-intensity demands. Unlike previous studies that focused on endurance-based sports such as cycling [9] or other combat sports such as judo [13], this research is the first to demonstrate the efficacy of IPC in taekwondo, a sport that requires rapid alternation between explosive anaerobic efforts and aerobic recovery. By employing the FSKT and PCr-La-O_2_ methods, we offer a detailed analysis of energy system contributions, thereby providing a sport-specific perspective on the metabolic benefits of IPC.

Our research indicates that IPC can enhance the performance of taekwondo athletes in specific tests. As the duration of exercise increases, the effect of IPC on performance becomes more pronounced, potentially achieved through the cumulative effect of IPC. Furthermore, IPC significantly increased the aerobic metabolic energy supply for taekwondo athletes during these specific tests. Moreover, marginally significant increases in the energy supply from the glycolytic system were observed, whereas no improvements were noted in the phosphagen system. Notably, the marginally significant increase in the energy supply of the glycolytic system may be related to an increase in blood lactate clearance rates.

In this study, our results indicate that IPC does not enhance motor performance during the first round (10 s) in the initial FSKT group. Previous research has confirmed that short-term high-intensity exercise lasting approximately 10 seconds relies primarily on the phosphagen system for energy supply [16], and this system can sustain high-intensity output for only up to 10 seconds. Therefore, the first round of the FSKT test is often used to assess the phosphagen system capacity of taekwondo athletes [22]. In this study, there was no significant difference in the phosphagen system energy supply between the IPC trial and the SHAM trial. This result aligns with several recent studies, indicating that IPC does not enhance the performance of 30-meter repeated sprints in team project athletes [41]. Furthermore, IPC fails to improve maximum sprint performance over distances of 10 meters or 20 meters in sprinters [42]. These sports rely primarily on the phosphagen energy supply. In contrast, previous research has shown that IPC reduces the ATP consumption rate during rest by inducing the opening of mitochondrial ATP-sensitive potassium ion channels [17,43]. Additionally, IPC can influence muscle activity by facilitating more effective muscle contractions. It promotes ATP retention by increasing the efficiency of excitation‒contraction coupling and reducing ineffective ion pumping. This may lead to improved muscle efficiency and a decrease in energy demand per unit of work [44], thereby increasing the preexercise ATP‒CP reserve. During intense exercise, the energy supply capacity of phosphagen predominantly depends on the reserve and resynthesis ability of ATP-CP [16,45]. These studies suggest that IPC may positively impact short-term intense exercise from the perspective of energy metabolism mechanisms. The observed contradictions may stem from factors related to energy supply, muscle coordination, muscle volume, and neural reflexes during short-term intense exercise [46–50]. Consequently, a mere increase in phosphagen reserves may not be sufficient to induce significant changes in exercise performance. Moreover, these contradictions may also be related to the duration of the exercise. The first round of FSKT in the first group had a movement duration that was too short, which limits the efficacy of IPC in sports primarily powered by the phosphagen system. Previous studies have reported similar findings [41]. Therefore, we conclude that IPC does not enhance the test results of the first round of FSKT in taekwondo athletes by increasing the energy supply of the phosphagen system.

Although some research has indicated that IPC can reduce lactate accumulation and enhance anaerobic glycolytic capacity during high-intensity exercise [15,16,44], as well as improve anaerobic activities such as 100-meter swimming, 400-meter running, and 30-second repeated sprints in cycling [7,8,51,52], our findings suggest that the energy supply from anaerobic glycolysis during IPC trials only marginally significantly improved. Furthermore, the total number of FSKT kicks, which reflects the specific anaerobic exercise capacity of taekwondo athletes, significantly improved, whereas lactate accumulation during the specialized testing process remained unchanged. This finding contradicts those of previous studies. During high-intensity exercise, the storage of phosphocreatine and the acid resistance ability of muscles are crucial determinants of anaerobic capacity, whereas the concentration of lactate serves as an indicator of the glycolytic supply [53]. However, some studies have indicated that following IPC in porcine skeletal muscle, lactate accumulation is reduced [44]. Concurrently, IPC has been shown to increase blood flow, which improves oxygen delivery and may facilitate the clearance of lactate [54,55]. These observations align with the results regarding lactate clearance rates in this study. Therefore, IPC may increase the body’s capacity to clear lactate, leading to a lower calculated accumulation of lactate; thus, no significant difference in the glycolytic energy supply is observed. Furthermore, research suggests that IPC can influence muscle activity by promoting more effective muscle contractions. This mechanism may enhance ATP retention by facilitating excitation‒contraction coupling and reducing ineffective ion pumping. Consequently, this may lead to improved muscle contraction efficiency and a decrease in energy requirements per unit of work [9,56]. Therefore, we propose that IPC may mitigate the detrimental effects of exercise-induced acidosis by increasing the lactate clearance rate and buffering capacity of the body [57]. Moreover, it enhances the capacity of anaerobic glycolysis to supply energy [16], thereby improving the anaerobic performance of taekwondo athletes. Additionally, IPC may also enhance muscle contraction efficiency, further increasing exercise performance.

Although the energy supply from glycolysis remains unchanged, the increased lactate clearance rate in the IPC indicates that aerobic metabolism may enhance the Cori cycle by providing the ATP required for gluconeogenesis, maintaining the dynamic balance between lactate and glucose, and supporting the function of lactate as an intertissue energy carrier, thereby promoting lactate reutilization [16,58]. IPC improves mitochondrial efficiency (such as the activation of mitochondrial ATP-sensitive potassium channels) by preventing the abnormal opening of the mitochondrial permeability transition pore (mPTP) during reperfusion, thereby maintaining the integrity of the mitochondrial membrane potential and avoiding interruptions in ATP synthesis. Moreover, it preserves the function of the electron transport chain, thereby reducing abnormal ATP consumption. Additionally, IPC reduces intracellular acidosis and delays the onset of fatigue [17,54,59,60]. This mechanism aligns with the intermittent nature of taekwondo, where rapid aerobic recovery between rounds is critical for maintaining phosphagen resynthesis [19].

We have conducted a relatively in-depth discussion of the impact of IPC on the energy supply of anaerobic metabolism. However, the most noteworthy aspect of this study is the positive effect of IPC on the aerobic energy supply capacity in special tests involving taekwondo athletes. Our results indicate that IPC significantly enhances the aerobic metabolic energy supply of taekwondo athletes, as evidenced by a marked increase in oxygen uptake during these special tests. This aligns with previous research findings. Several studies have demonstrated that IPC can increase the maximum oxygen uptake in cycling athletes by 3% [9] and improve 5-kilometer running performance in healthy males [61], suggesting that IPC has a beneficial effect on aerobic exercise [62]. Furthermore, some studies indicate that IPC can induce the endogenous production of adenosine, bradykinin, nitric oxide, and opioid peptides [59,63] and activate mK_ATP_ [17,64]. Elevated mK_ATP_ and adenosine levels can promote vasodilation [9,54] and enhance muscle blood flow [55]. Additionally, IPC can regulate microvascular dilation via nitric oxide, exert anti-inflammatory effects, improve vascular function during exercise, increase oxygen supply efficiency, facilitate lactate clearance, and maintain acid‒base equilibrium [55,65]. Moreover, certain studies suggest that IPC enables mitochondria to take up acetyl-CoA more rapidly, thereby maintaining lactate accumulation within metabolically tolerable limits and contributing to aerobic ATP production during physical activity [15]. These findings corroborate the results of the present study, which indicates that IPC can increase aerobic metabolism. Therefore, we propose that IPC can improve the aerobic metabolic energy supply for taekwondo athletes. We further discuss the results in conjunction with specific tests. Research indicates that when exercise duration exceeds approximately one minute (as seen in the 800-meter track and field event), oxidative phosphorylation becomes the primary method for ATP production [66]. As exercise duration increases, the contribution of aerobic metabolism to the energy supply continually increases [16]. This aligns with the results of exercise testing, where the longer the duration of exercise, the stronger the cumulative effect of IPC in enhancing aerobic metabolism, leading to a greater cumulative improvement in athletic performance. Our findings also demonstrated that in the IPC trial, exercise performance in specific tests significantly improved compared with that in the SHAM trial, particularly during the middle and later stages of the test. This aligns with previous studies that reported enhanced exercise performance in the 3-minute bicycle all-out test due to IPC [67]. Thus, we propose that as the proportion of aerobic energy supply increases, the benefits of IPC on exercise performance become more pronounced. Furthermore, research has shown that aerobic ATP production can be activated even during very intense exercise. During the final five seconds of a 30-second all-out sprint, approximately 50% of the energy is derived from aerobic metabolism [16]. This may account for the notable improvements in exercise performance and total punching output in the first round of the third group of FSKT tests, despite the lack of significant increases in phosphagen and glycolytic energy supplies. Notably, we also observed a marginally significant increase in accumulated oxygen uptake during high-intensity FSKT tests in the third set. This may be attributed to IPC’s enhancement of vascular function, which prevents the decline in vascular function following high-intensity exercise [68,69], thereby improving aerobic capacity during such exercise.

Overall, the application of IPC significantly improved the performance of taekwondo athletes in their specific tests by increasing their aerobic metabolic capacity in the second group of FSKT tests and the third group of FSKT tests. Concurrently, the observed increase in accumulated oxygen uptake and the elevated blood lactate clearance rate indicate an increase in aerobic metabolism. This improvement effectively facilitates the removal of lactate, thereby preventing its premature accumulation and subsequent acidosis, which contributes to delaying fatigue and maintaining a stable and efficient energy supply [16]. Consequently, this process reduces reliance on anaerobic glycolysis and enhances the synergistic relationship between glycolysis and aerobic metabolism, ultimately leading to improved sports performance. However, further research is needed to fully understand the impact of IPC on the function of the phosphagen system.

Several limitations should be noted. First, participants were aware of the cuff pressure differences between the IPC and sham trials, meaning that the study was not fully blinded. Second, our method of estimating the contribution of glycolytic energy (based on lactate accumulation) might have underestimated actual glycolytic output under IPC conditions since IPC accelerated lactate clearance. This limitation could explain why the difference in glycolytic energy only approached significance (p = 0.055) despite improved performance. Third, the sample size (n = 16) provided adequate power to detect moderate-to-large performance effects, but it was likely underpowered for smaller changes in secondary measures such as the glycolytic energy supply. A larger cohort might reveal additional subtleties, such as slight phosphagen system effects or more robust statistical confirmation of the trends we observed. Future research with larger samples and rigorous blinding is needed to validate our findings and to further elucidate IPC’s impact across all energy systems in combat sports.

## Conclusions

The application of 40 minutes of IPC to the lower limbs 20 minutes before exercise can enhance the specific performance of Taekwondo athletes. This may have a positive effect on the competition and training performance of Taekwondo practitioners. This technique significantly improves aerobic metabolic capacity and optimizes its synergistic effect with the glycolytic energy supply. IPC has clear practical value and application prospects in Taekwondo training and competition.

## Supporting information

S1 File CONSORT 2025 checklist. (PDF)

S2 File Experimental protocol. (PDF)

S3 File Experimental protocol translated. (PDF)
