# Supplementary material for: Ischemic preconditioning enhances energy supply during frequency speed kick test in Taekwondo athletes: A randomized crossover study
Source: PLoS One. 2026 Feb 3;21(2):e0341780. doi: 10.1371/journal.pone.0341780 (PMC12867267; doi:10.1371/journal.pone.0341780)
Supplement: S1 File — (PDF) [file pone.0341780.s001.pdf]

CONSORT 2025 checklist

Section/topic	No	CONSORT 2025 checklist item description	Reported on page no.
Title and abstract			
Title and structured abstract	1a	Identification as a randomised trial	1 Title (indicates "Randomized Crossover Study")
	1b	Structured summary of the trial design, methods, results, and conclusions	1 (Abstract)
Open science			
Trial registration	2	ClinicalTrials.gov ID: NCT07170774	5 (Methods: Subjects)
Protocol and statistical analysis plan	3	Protocol and statistical analysis plan: Randomized Crossover Controlled Trial, shapiro-Wilk test, two-way repeated measures ANOVA, generalized linear mixed models.	5 (Methods)<br>11-13 (Data analysis)
Data sharing	4	Where and how the individual de-identified participant data can be accessed	24 (Data availability section)
Funding and conflicts of interest	5a	Sources of funding and other support	24, “Funding” section
	5b	Financial and other conflicts of interest of the manuscript authors	24, “Competing interests” section
Introduction			
Background and rationale	6	Scientific background and rationale	2, Introduction section
Objectives	7	Specific objectives related to benefits and harms	2, Introduction section
Methods			
Patient and public involvement	8	Details of patient or public involvement in the design, conduct and reporting of the trial	5, “Methods” section
Trial design	9	Description of trial design including type of trial, allocation ratio, and framework	5, “Methods” section

Section/topic	No	CONSORT 2025 checklist item description	Reported on page no.
Changes to trial protocol	10	Important changes to the trial after it commenced	5-7, “Methods” section
Trial setting	11	Settings and locations where the trial was conducted	5-7, “Methods” section
Eligibility criteria	12a	Eligibility criteria for participants	5, “Subjects” section
	12b	Eligibility criteria for sites and individuals delivering the interventions	Not applicable
Intervention and comparator	13	Intervention and comparator with sufficient details to allow replication	8-9, “IPC” section
Outcomes	14	Prespecified primary and secondary outcomes	8-11, “Methods” section
Harms	15	How harms were defined and assessed	Not applicable
Sample size	16a	How sample size was determined	4, “Subjects” section
	16b	Explanation of any interim analyses and stopping guidelines	Not applicable
Randomisation			
Sequence generation	17a	Random sequence generation: an independent statistician A, who was not involved in the recruitment of subjects, generated the allocation sequence using R software (version 4.3.2).	6, “Methods” section
	17b	Type of randomization: a block randomization design was employed (with a block length of 4) to ensure a balanced distribution of the two intervention sequences (A-B or B-A) throughout the recruitment period. A random seed number (20240904) was set to ensure reproducibility.	6, “Methods” section
Allocation concealment mechanism	18	Allocation concealment: we strictly implemented allocation concealment using the Sequentially Numbered, Opaque, Sealed Envelope (SNOSE) method. The envelopes were prepared by a research assistant B and were kept by another research assistant C. For each eligible subject recruited, the researcher contacted research assistant C to open the next sequential envelope and learn the assigned sequence.	6, “Methods” section
Implementation	19	Implementation: Staff administering intervention were aware of pressure	6, “Methods” section
Blinding	20a	Blinding: Single-blind (outcome assessors blinded)	6, “Methods” section

Section/topic	No	CONSORT 2025 checklist item description	Reported on page no.
	20b	Similarity of interventions: Pressure difference made participant blinding impossible	6, “Methods” section
Statistical methods	21a	Statistical methods: Repeated-measures ANOVA, GLMM, etc.	11-13, “Data analysis” section
	21b	Analysis population: All 16 participants included	13, “Data analysis” section
	21c	Missing data: None	None
	21d	Methods for any additional analyses	11-13, “Data analysis” section
Results			
Participant flow, including flow diagram	22a	For each group, the numbers of participants who were randomly assigned, received intervention, and analysed	Implied (all 16 completed)
	22b	For each group, losses and exclusions after randomisation, together with reasons	None
Recruitment	23a	Dates defining the periods of recruitment and follow-up	5, “Subjects” section
	23b	Trial end reason: Participants may withdraw from the trial at any time.	5, “Subjects” section
Intervention and comparator delivery	24a	Intervention and comparator as they were actually administered	8, “IPC” section
	24b	Concomitant care received during the trial for each group	Not applicable
Baseline data	25	A table showing baseline demographic and clinical characteristics for each group	13, Table 1
Numbers analysed, outcomes and estimation	26	For each primary and secondary outcome, by group: number analysed, result, effect size	13-18, Results section
Harms	27	All harms or unintended events in each group	None
Ancillary analyses	28	Any other analyses performed, including subgroup and sensitivity analyses	None
Discussion			

Section/topic	No	CONSORT 2025 checklist item description	Reported on page no.
Interpretation	29	Interpretation consistent with results, balancing benefits and harms	18-23, Discussion section
Limitations	30	Trial limitations, addressing sources of potential bias, imprecision, generalisability	23, Discussion section

Citation: Hopewell S, Chan AW, Collins GS, Hróbjartsson A, Moher D, Schulz KF, et al. CONSORT 2025 Statement: updated guideline for reporting randomised trials. BMJ. 2025; 388:e081123. https://dx.doi.org/10.1136/bmj-2024-081123

© 2025 Hopewell et al. This is an Open Access article distributed under the terms of the Creative Commons Attribution License (https://creativecommons.org/licenses/by/4.0/), which permits unrestricted use, distribution, and reproduction in any medium, provided the original work is properly cited.

*We strongly recommend reading this statement in conjunction with the CONSORT 2025 Explanation and Elaboration and/or the CONSORT 2025 Expanded Checklist for important clarifications on all the items. We also recommend reading relevant CONSORT extensions. See www.consort-spirit.org.
