# Supplementary material for: Ischemic preconditioning enhances energy supply during frequency speed kick test in Taekwondo athletes: A randomized crossover study
Source: PLoS One. 2026 Feb 3;21(2):e0341780. doi: 10.1371/journal.pone.0341780 (PMC12867267; doi:10.1371/journal.pone.0341780)
Supplement: S2 File — (PDF) [file pone.0341780.s002.pdf]

干预方案与实验流程

实验参与者将接受两次相同的测试流程，其中一次包含缺血预处理，而另一次则安慰剂干预。在这两次测试中，唯一的区别在于施加于腿部的压力强度。在进行缺血预处理的测试中，将对大腿施加 220mmHg 的压力。据研究显示，当对大腿（中上三分之一处）施加 220mmHg 的压力时，可以有效地造成腿部局部缺血。实验流程如下：

（1）基本资料调查，姓名、年龄、身高、体重、训练时长和运动员等级水平等基本资料；

（2）讲解实验流程和熟悉踢击流程；

（3）穿戴运动心肺功能测定仪（JAEGER，Oxycon Mobile，德国）、加压设备（Theratools，BFR 下肢，中国）和心率带（博能，Polar H10），佩戴运动心肺功能测定仪前需对设备进行至少 30 分钟预热并通过校准调试，加压设备穿戴位置为大腿中上三分之一部位，心率带佩戴于胸前。穿戴完成后，静坐 5min，结束后使用电子血压仪测量血压（OMRON 上臂式，HEM-1020）；

（4）实验对象平躺，询问主观运动疲劳感觉（RPE），测量血氧（PC-60C），加压开始前采指尖血（EKF，德国），使用 Biosen C_Line 葡萄糖/乳酸自动分析仪（EKF，德国）测量血乳酸；

（5）开启运动心肺功能测试仪记录全过程的气体数据和开启运动软件 POLAR FLOW 记录全程心率数据；

（6）开始对大腿施加压力，每次缺血处理 5min，左右腿交替进行共 10 分钟为一组，进行四组 40 分钟，压力值为 220mmHg，组成缺血预处理。非缺血预处理测试则需要对大腿施加 20mmHg 压力，流程如缺血预处理测试；

（7）干预结束后，休息 5min；测量血氧、RPE 和采集休息第 3min 时的指尖血；

（8）休息后进行 10min 热身活动，由 5min 慢跑（心率控制在 120 次/分钟左右）、3 组 20 秒中等强度（心率控制在 140-160 次/分钟）左右横踢和柔韧拉伸组成，左右横踢的模式与跆拳道专项无氧能力测试（FSKT）相似，让实验对象尽量熟悉运动测试流程。热身后休息 5min，休息过程中测量 RPE、血氧和采集运动开始前即刻的指尖血；

（9）休息结束后开始三组跆拳道专项无氧能力测试（FSKT），FKST 采用 WT 跆拳道大满贯电子护具（专业版）和人形立式沙包进行测试，电脑记录踢击的次数。完整

测试时间为 6 分 30 秒，每组 FSKT 测试 90s，每组间歇时间为 60s，与跆拳道竞技比赛赛制相似（三局比赛，局间休息 1 分钟），每次间歇时间询问运动员 RPE；

（10）运动测试结束第 3 和 10 和 15 分钟采集指尖血，运动测试后即刻、5 分钟、10 分钟、15 分钟询问 RPE，运动后即刻测量血氧；

（11）实验结束。

缺血预处理流程如下图 1

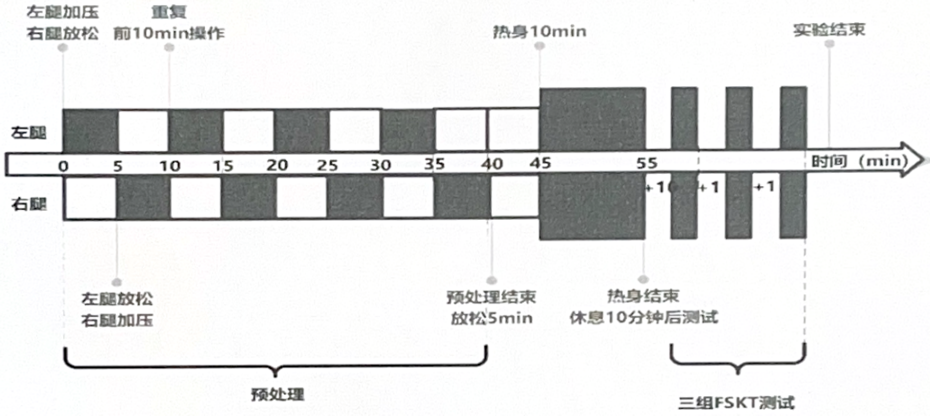

图 1 缺血预处理流程和测试流程图

完整实验流程如下图 2

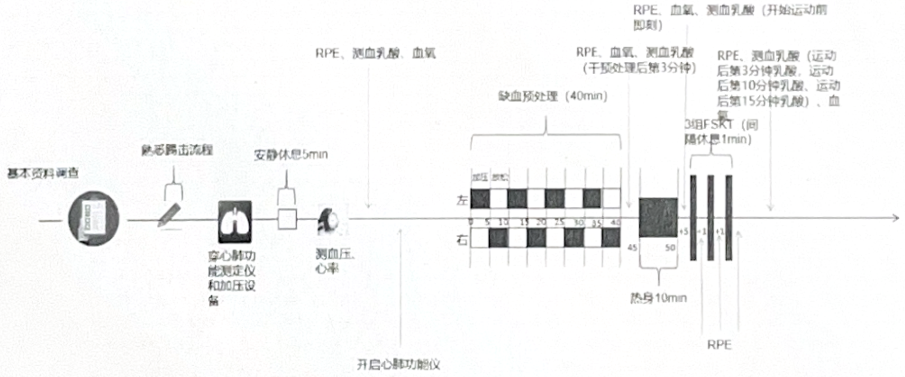

图 2 实验完整流程图
