# Supplementary material for: Ischemic preconditioning enhances energy supply during frequency speed kick test in Taekwondo athletes: A randomized crossover study
Source: PLoS One. 2026 Feb 3;21(2):e0341780. doi: 10.1371/journal.pone.0341780 (PMC12867267; doi:10.1371/journal.pone.0341780)
Supplement: S3 File — (PDF) [file pone.0341780.s003.pdf]

Intervention Plan and Experimental Process

Participants will undergo two identical test protocols, with one session featuring ischemic preconditioning and the other receiving placebo intervention. The sole difference between these trials lies in the applied leg pressure intensity. During the ischemic preconditioning trial, 220mmHg pressure is applied to the thigh. Research indicates that applying 220mmHg pressure to the upper-middle third of the thigh effectively induces localized leg ischemia. The experimental protocol is as follows:

(1) Basic information survey, name, age, height, weight, training duration and athlete level and other basic information;

(2) Explain the experimental process and familiarize yourself with the kicking process;

(3) Wear the exercise cardiopulmonary function tester (JAEGER, Oxycon Mobile, Germany), compression device (Theratools, BFR Lower Limb, China), and heart rate strap (Bon, Polar H10). Before wearing the exercise cardiopulmonary function tester, warm up the equipment for at least 30 minutes and calibrate it. The compression device should be worn on the upper-middle third of the thigh, while the heart rate strap is worn around the chest. After completing the wear, sit quietly for 5 minutes. Finally, use an electronic blood pressure monitor (OMRON Arm-type, HEM-1020) to measure blood pressure.

(4) The subjects were lying down, asked about subjective exercise fatigue (RPE), measured blood oxygen (PC-60C), collected fingertip blood (EKF, Germany) before the start of pressurization, and used Biosen C Line glucose/lactic acid automatic analyzer (EKF, Germany) to measure blood lactate;

(5) Start the exercise cardiopulmonary function tester to record the whole process of gas data and start the exercise software POLAR FLOW to record the whole process of heart rate data;

(6) The ischemic preconditioning protocol involved applying pressure to the thigh for 5 minutes per session, alternating between both legs with each session lasting 10 minutes. This was repeated four times for a total of 40 minutes, maintaining a pressure level of 220 mmHg. The non-ischemic preconditioning test required applying 20 mmHg pressure to the thigh, following the same procedure as the ischemic preconditioning test.

(7) After the end of the intervention, rest for 5min; measure blood oxygen, RPE and collect fingertip blood at the 3rd minute of rest;

(8) After resting, participants performed a 10-minute warm-up consisting of 5 minutes of jogging (heart rate controlled at approximately 120 beats per minute) followed by three sets of 20-second moderate-intensity side kicks (heart rate maintained between 140-160 bpm) and flexibility stretches. The side kick pattern mimicked the Taekwondo-specific anaerobic capacity test (FSKT), helping subjects familiarize themselves with the exercise protocol. Following the warm-up, a 5-minute rest period was observed during which researchers measured perceived exertion (RPE), blood oxygen levels, and collected fingertip blood samples immediately before exercise initiation.

(9) After the break, three sets of Taekwondo-specific anaerobic capacity tests (FSKT) were conducted. The FKST used the WT Taekwondo Grand Slam Electronic Protective Gear (Professional Edition) and a humanoid vertical punching bag for testing, with the computer recording the number of kicks.

The test time was 6 minutes and 30 seconds, each FSKT test lasted 90 seconds, and the interval time between each group was 60 seconds, which was similar to the format of taekwondo competitive competition (three rounds, one minute rest between rounds), and the RPE of athletes was asked during each interval time;

(10) Finger blood was collected at the end of exercise test at 3, 10 and 15 minutes. RPE was asked immediately, 5 minutes, 10 minutes and 15 minutes after exercise test. Blood oxygen was measured immediately after exercise;

(11) The experiment is over.

The ischemic pre-treatment process is shown in Figure 1

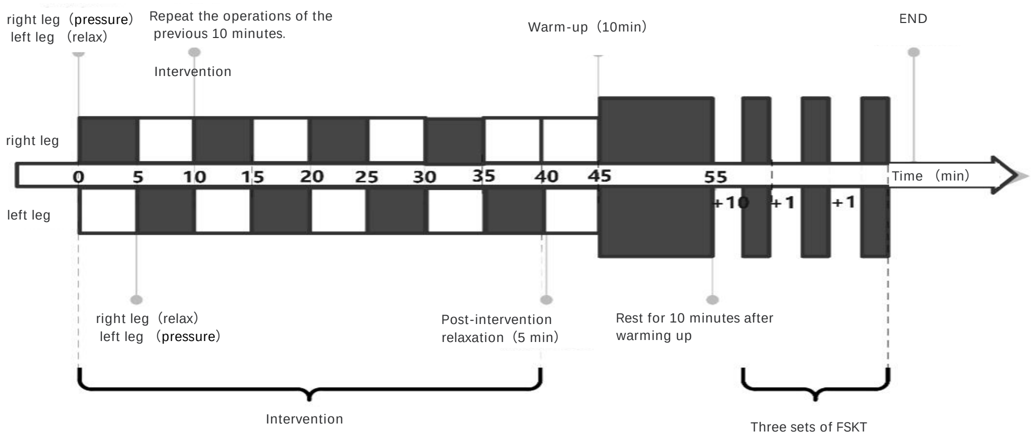

Figure 1 Flow Chart of Ischemic Pre-Treatment and Test

The complete experimental process is shown in Figure 2

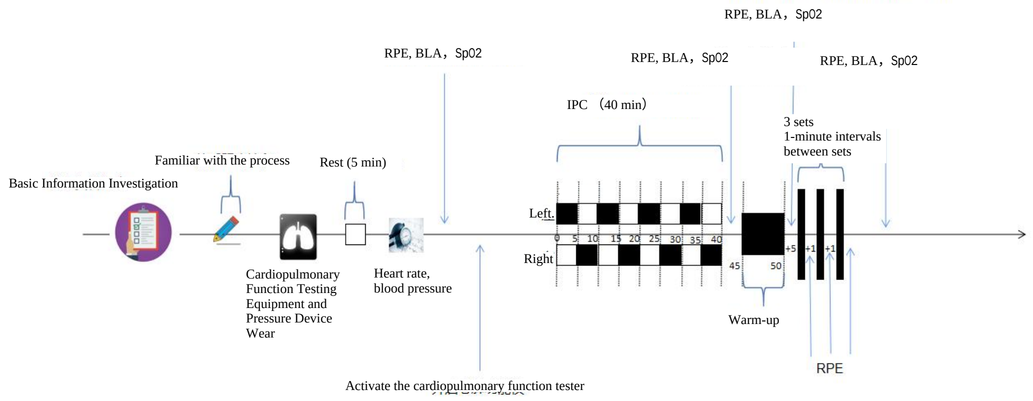

Figure 2 Complete Flow Chart of the Experiment
